# Investigation of the effect of vitamin K1 prophylaxis on newborn screenings tests in newborns

**DOI:** 10.5937/jomb0-40162

**Published:** 2023-08-25

**Authors:** Murat Caglayan, Ataman Gonel, Cuneyt Tayman, Ufuk Cakir, Ismail Koyuncu, Ebru Temiz, Yasemin Sert

**Affiliations:** 1 Health Science University, Diskapi Yildirim Beyazit Training and Research Hospital, Department of Medical Biochemistry, Ankara, Turkey; 2 Hasan Kalyoncu University, Faculty of Health Science, Medical Park Gaziantep Hastanesi, Department of Nutrition and Dietetics, Gaziantep, Turkey; 3 University of Health Sciences, Ankara City Hospital, Department of Neonatology, Cankaya, Ankara, Turkey; 4 Harran University, Medicine Faculty, Department of Medical Biochemistry, Sanliurfa, Turkey; 5 Harran University, Health Services Vocational School, Program of Medical Promotion and Marketing, Sanliurfa, Turkey; 6 Ankara City Hospital, Department of Gynecology Obstetrics, Ankara, Turkey

**Keywords:** Vitamin K1, heel blood, hereditary disorders, tandem MS, interference, newborn, vitamin K1, krv pete, nasledni poremećaji, tandem MS, interferencija, novorođenče

## Abstract

**Background:**

Routine screening for hereditary disorders in newborns includes screening for treatable metabolic and endocrine disorders, such as biotidinase deficiency, galactosemia, maple syrup urine disease, hypothyroidism, and cystic fibrosis. Incorrect test results may be encountered due to the use of vitamin K1. To investigate the interference effect of vitamin K1 on neonatal screening tests and to raise awareness of erroneous measurements.

**Methods:**

Heel blood samples were taken from 25 newborns born in a neonatal intensive care unit. Dry blood C0, C2, C3, C4, C4DC, C5:1, C5OH, C5DC, C6, C6DC, C8, C8:1, C8DC, C10, C10:1, C10DC, C12, C14, C14:1, C14:2, C16, C16:1, C18, C18:1, C18:2, C18:OH, methylglutaryl, valine, leucine/isoleucine, methionine, phenylalanine, argininosuccinic acid, aspartate, alanine, arginine, citrulline, glycine, ornithine, and glutamate tests were studied using the tandem mass spectrometry (MS) method. The results of the heel blood samples obtained before and after the application of vitamin K1 (Phyto menadione) were compared.

**Results:**

In two studies conducted with in vitro and in vivo tests, C0, C2, C3, C4, C4DC, C5, C5OH, C6, C8, C10, C10:1, C14, C16, C16:1, C18, C18:1, methylglutaryl, phenylalanine, argininosuccinic acid, tyrosine, aspartate, arginine, citrulline, glycine, and glutamine were all significantly elevated (p < 0.05).

**Conclusions:**

Heel blood samples may yield false results due to vitamin K1 administration. In the case of doubtful results, a new sample should be taken and the measurement should be repeated.

## Abstract (sr)

**Uvod:**

Rutinski skrining za nasledne poremećaje kod novorođenčadi uključuje skrining metaboličkih i endokrinih poremećaja koji se mogu lečiti, kao što su nedostatak biotidinaze, galaktozemija, bolest urina od javorovog sirupa, hipotiroidizam i cistična fibroza. Mogu se naći i netačni rezultati zbog upotrebe vitamina K1. Cilj je bio da se istraži efekat interferencije vitamina K1 na neonatalne skrining testove i da se podigne svest o pogrešnim merenjima.

**Metode:**

Uzorci krvi iz pete uzeti su od 25 novorođenčadi rođenih u neonatalnoj jedinici intenzivne nege. Suva krv C0, C2, C3, C4, C4DC, C5:1, C5OH, C5DC, C6, C6DC, C8, C8:1, C8DC, C10, C10:1, C10DC, C12, C14, C14:1, C14: 2, C16, C16:1, C18, C18:1, C18:2, C18:OH, metilglutaril, valin, leucin/izoleucin, metionin, fenilalanin, ar ginin osukcinska kiselina, aspartat, alanin, arginin, citrulin, glicin, ornitin i glutamat testovi su proučavani metodom tandem masene spektrometrije (MS). Upoređeni su rezultati uzoraka krvi pete pre i posle primene vitamina K1 (Phito menadion).

**Rezultati:**

U dve studije sprovedene sa in vitro i in vivo testovima, C0, C2, C3, C4, C4DC, C5, C5OH, C6, C8, C10, C10:1, C14, C16, C16:1, C18, C18: 1, metilglutaril, fenilalanin, argininosukcinska kiselina, tirozin, aspartat, arginin, citrulin, glicin i glutamin koji su svi značajno bili povišeni (p < 0,05).

**Zaključak:**

Uzorci krvi pete mogu dati lažne rezultate zbog davanja vitamina K1. U slučaju sumnjivih rezultata, treba uzeti novi uzorak i ponoviti merenje.

## Introduction

Routine screening for hereditary disorders in newborns began in 1960, when Robert Guthrie introduced a simple bacterial inhibition test to detect high concentrations of phenylalanine in dried blood samples [1]. Newborn screening has since been expanded to include treatable metabolic and endocrine disorders, such as biotidinase deficiency, galactosemia, maple syrup urine disease, hypothyroidism, and cystic fibrosis. A method of screening for metabolic diseases with a broad-spectrum metabolic profile obtained from a single sample using mass spectrometry (MS) was defined and developed in 1990 [1] [2]. This method has greatly changed newborn screening, as it provides rapid, simultaneous analysis of multiple analytes, allowing for the identification of more than 40 disorders of amino, organic, and fatty acid metabolism [3]. Mass spectrometers take measurements based on the differentiation of charged particles by their movement within an electrical or magnetic field, in accordance with their mass and charge ratios [4]. Although the number of diseases diagnosed through newborn screening is significant, there is a high incidence of false-positive test results. This results in retest costs and psychological effects on parents. Given that tandem MS devices measure metabolites at very low rates, all kinds of endogenous and exogenous substances in the blood matrix can change the density of the matrix, resulting in incorrect measurements (the matrix effect) [5]. Excessive consumption of biotin (5–100 mg per day) taken as a supplement poses a significant problem for clinical immunological tests based on biotin–streptavidin interactions. It may not be possible to determine how many patients have been affected by biotin-induced erroneous results in the past. However, by showing in advance the quantities of drugs required to have such effects on certain tests, malpractice can be prevented in the future. Incorrect test results may also be encountered due to the use of vitamin K1, such as biotin, in newborns. There are currently no data on how vitamin K1 affects newborn screening tests. Therefore, the aims of this study are to investigate the interference effect of vitamin K1 on neonatal screening tests and to raise awareness about erroneous measurements.

## Materials and methods

Heel blood samples were taken from 25 newborns born in the neonatal intensive care unit of Zekai Tahir Burak Women's Health Education and Research Hospital after delivery. Then, a second heel blood sampling was conducted 12 hours after the administration of vitamin K1 (Phyto menadione; Libavit K ampoule, 2 mg in 0.2 mL, Mefar Drug Company, Istanbul, Turkey). Newborns with suspected premature birth, active infection, metabolic acidosis, genetic and major congenital anomalies, or metabolic defects were not included in the study. Dry blood C0, C2, C3, C4, C4DC, C5:1, C5OH, C5DC, C6, C6DC, C8, C8:1, C8DC, C10, C10:1, C10DC, C12, C14, C14:1, C14:2, C16, C16:1, C18, C18:1, C18:2, C18:OH, methylglutaryl, valine, leucine/isoleucine, methionine, phenylalanine, argininosuccinic acid, aspartate, alanine, arginine, citrulline, glycine, ornithine, and glutamate were tested using the tandem MS method (Shimadzu 8040, Japan). The results of the heel blood samples taken before and after the application of vitamin K1 (Phyto menadione) were compared for all eligible patients. 10 uL of vitamin K1 (Libavit K) (with 1/10 dilution) was dripped onto the sample taken before vitamin K1 was administered to the samples in the study group for repeat measurement. The measurement was performed three times, and the average values were recorded. The mean NBS (C0, C2, C3, C4, C4DC, C5:1, C5OH, C5DC, C6, C6DC, C8, C8:1, C8DC, C10, C10:1, C10DC, C12, C14, C14:1, C14:2, C16, C16:1, C18, C18:1, C18:2, C18:OH, methylglutaryl, valine, leucine/isoleucine, methionine, phenylalanine, argininosuccinic acid, aspartate, alanine, arginine, citrulline, glycine, ornithine, and glutamate) values obtained before and after in vitro vitamin K1 instillation were compared.

### Materials and reagents

High-performance liquid chromatography (HPLC)-rated formic acid was purchased from Fisher Scientific (Loughborough, UK). The water used in the experiments was obtained through a Milli-Q ultrapure water system (Millipore, Billerica, MA). The Agilent ZORBAX ODS C18 column (Agilent Technologies, Santa Clara, CA; 150 mm, 2.1 mm, 3.5 mm) was used for all analyses.

### Sample preparation

Sample preparation for the routine screening tests was performed exactly as reported by LaMarca et al. [6] in 2008, with the exception of the butylation procedure, which was not carried out. The carnitine profile was studied by modifying the newborn screening method developed by LaMarca et al. [6] and Azzari et al. [7]. Filter papers cut into 3.2-mm discs (Whatman filter paper 10538018) were placed onto plates of 96. A 5-μL plasma sample was added to it and dried at room temperature overnight.

### Tandem mass spectrometry

The sample was extracted by dispensing 300 μL of an extraction solution consisting of a mixture of methanol and an aqueous solution of 3 mmol/L hydrate hydrazine at approximate relative volume/volume ratios of 66.6% and 33.3%, respectively. Internal standards, stable heavy isotope analogues of several amino acids, carnitine, and acylcarnitines were also present in the extract solution. The extracted sample was injected into a Shimadzu LCMS-8040. Mass spectral data for the amino acids were acquired through a neutral loss scan of 46 Da in positive mode (CE-15V), while mass spectral data for the acylcarnitines were acquired through a precursor ion scan of 85 m/z in positive mode (CE-25V). The percentage of each analyte recovered was determined through comparison with an internal standard. The standard concentrations of the acylcarnitines fell within the range of 7.6-152 μmol/L. Samples spiked with different concentrations of analytes were used for a daily quality control test.

### Analysis condition

Run of 2.2 minutes in flow injection analysis (FIA), flow 0.070 μL/min (A: water + 0.05% formic acid, B: acetonitrile, A/B: 30%/70%); 40 μL of sample injected, column oven 30°C, desolvatation line 300°C, heat 500°C, nebulizing gas 3 L/min, drying gas 20 L/min. All data were reprocessed using Shimadzu Neonatal Software, which automatically calculated the concentration of each compound.

### Statistical analysis

A Shapiro–Wilk test was used to determine the suitability of the data for normal distribution. Based on the results of the Shapiro–Wilk test, a Student's t-test was used for those that showed a normal distribution within their groups (p > 0.05), and a Mann-Whitney U test was used for those that did not show a normal distribution (p < 0.05). The deficiency ratios between the mean values were calculated using bias. GraphPad Prism 6 (GraphPad Software, Inc., San Diego, USA) was used for statistical analysis, and p < 0.05 was considered statistically significant.

## Results

The demographic data of the patients are summarized in Table 1. In two studies conducted with in vitro and in vivo tests, C0, C2, C3, C4, C4DC, C5, C5OH, C6, C8, C10, C10:1, C14, C16, C16:1, C18, C18:1, methylglutaryl, phenylalanine, argininosuccinic acid, tyrosine, aspartate, arginine, citrulline, glycine, and glutamine were all significantly elevated. Although C10DC, C12, and C14:1 significantly increased in the in vivo study, these values were not significantly affected in the in vitro study. Similarly, while C5DC, C6DC, C8DC, C14:2, and C18:1 significantly increased in the in vivo study, these values significantly decreased in the in vitro study. Conversely, C18:2, valine, and leucine decreased in the in vivo study but increased in the in vitro study. The observed increases in C5:1, C8:1, methionine, ornithine, and alanine were not significant in the in vivo study but were significant in the in vitro study. Deviants between -14.15% and 166.67% occurred in the in vivo study. The most deviant test was the C14:1 test (166.67%), while the least deviant test was the alanine test (2.26%; Table 2).

**Table 1:** Demographic and clinical features of the patients

Variables	Patients (n=25)
Maternal age, years	29 (19–40)
Gestational age, weeks	38.2±1.88
Birth weight, g	3360±535
Male gender, n, (%)	13 (52)
Mode of delivery (Vaginal), n, (%)	18 (72)
Apgar score 1st min, a	8 (1)
Apgar score 5th min, a	9 (1)

**Table 2:** Analysis of samples in vivo and in vitro by Tandem mass spectrometry (LC-MS/MS)

	In vivo Study (n:25)						In vitro Study (n:20)					
Tests	Before<br>Vitamin K<br>(μ mol/L)	After<br>Vitamin K	Status	Difference	%<br>Bias	P<br>Value	Before<br>Vitamin K	After<br>Vitamin K<br>(μ mol/L)	Status	Difference	%<br>Bias	P<br>value
C0	15.84±5.08	20.68±5.51	↑	4.84	30.56	0.002	21.42±0.32	33.91±0.87	↑	12.49	58.31	0.05
C2	9.03±3.53	12.7±3.1	↑	3.66	40.58	<0.001	12.26±0.18	28.2±0.72	↑	15.94	130.02	0.05
C3	1.23±0.44	2.22±0.76	↑	0.98	80.19	<0.001	0.58±0.008	1.24±0.03	↑	0.66	113.79	0.05
C4	0.15±0.08	0.25±0.13	↑	0.09	59.55	0.005	0.05±0.007	0.12±0.003	↑	0.07	140	0.05
C4DC	0.14±0.05	0.18±0.06	↑	0.04	35.14	0.004	0.18±0.002	0.24±0.006	↑	0.06	33.33	0.05
C5	0.14±0.03	0.17±0.03	↑	0.02	20.34	0.007	0.04±0.0006	0.08±0.002	↑	0.03	96.56	0.05
C5:1	0.34±0.1	0.39±0.08	↑	0.04	13.72	0.08	0.02±0.0003	0.02±0.0005	↑	0.0003	1.48	0.37
C5OH	0.20±0.05	0.24±0.05	↑	0.04	20.12	0.011	0.081±.001	0.14±0.003	↑	0.06	77.37	0.05
C5DC	0.11±0.02	0.17±0.04	↑	0.06	60	<0.001	0.04±.0006	0.03±0.0007	↓	-0.009	-24.08	0.05
C6	0.03±0.007	0.04±0.01	↑	0.01	44.74	<0.001	0.01±.0001	0.02±0.0005	↑	0.01	101.96	0.05
C6DC	0.11±0.03	0.13±0.02	↑	0.02	19.35	0.02	0.04±0.0006	0.02±0.0005	↓	-0.02	-49.39	0.05
C8	0.03±0.01	0.05±0.02	↑	0.01	48.84	0.01	0.01±0.0001	0.02±0.0005	↑	0.01	101.96	0.05
C8:1	0.16±0.12	0.21±0.17	↑	0.04	27.88	0.28	0.01±0.0001	0.03±0.0007	↑	0.02	202.94	0.05
C8DC	0.11±0.03	0.15±0.03	↑	0.04	43.32	<0.001	0.05±0.0007	0.02±0.0005	↓	-0.03	-59.45	0.05
C10	0.03±0.01	0.06±0.01	↑	0.03	106.67	<0.001	0.01±0.0001	0.03±0.0007	↑	0.02	202.94	0.05
C10:1	0.30±0.09	0.36±0.09	↑	0.06	19.87	0.02	0.02±0.0003	0.04±.001	↑	0.02	102.96	0.05
C10DC	0.08±0.02	0.13±0.02	↑	0.05	59.91	<0.001	0.03±0.0004	0.03±0.0007	↑	0.0004	1.31	0.37
C12	0.06±0.03	0.14±0.05	↑	0.08	131.68	<0.001	0.03±0.0004	0.03±0.0007	↑	0.0004	1.31	0.37
C14	0.12±0.05	0.25±0.09	↑	0.13	108.67	<0.001	0.06±0.0009	0.07±0.001	↑	0.01	18.2	0.05
C14:1	0.05±0.04	0.13±0.06	↑	0.08	166.67	<0.001	0.03±0.0004	0.03±.0007	↑	0.0004	1.31	0.37
C14:2	0.13±0.02	0.16±0.03	↑	0.02	20.18	0.004	0.08±0.001	0.06±.001	↓	-0.01	-23.99	0.05
C16	1.65±0.6	2.78±0.62	↑	1.13	68.42	<0.001	0.66±0.009	0.91±.023	↑	0.25	38.73	0.05
C16:1	0.51±0.27	1.21±0.33	↑	0.69	135.11	<0.001	0.11±0.001	0.13±.003	↑	0.02	19.77	0.05
C18	0.58±0.17	0.8±0.17	↑	0.22	38.18	0.001	0.19±0.002	0.28±.007	↑	0.09	49.28	0.05
C18:1	1.86±0.76	3.24±0.77	↑	1.38	73.95	<0.001	0.13±0.001	0.2±.005	↑	0.07	55.82	0.05
C18:2	0.73±0.26	0.66±0.23	↓	-0.06	-8.94	0.36	0.08±0.001	0.18±.004	↑	0.1	128.04	0.05
C18:1	0.01±0.005	0.02±0.005	↑	0.01	63.16	<0.001	0.02±0.0003	0.01±.0002	↓	-0.01	-49.26	0.05
Methyl<br>Glutaryl	0.11±0.05	0.20±0.05	↑	0.09	89.09	<0.001	0.01±0.0001	0.02±.0005	↑	0.0104	101.96	0.05
Val	83.53±15.76	71.71±14.26	↓	-11.82	-14.15	0.008	76.48±1.14	143.84±3.69	↑	67.36	88.09	0.05
Leu\Ile	82.69±19.52	71.89±14.38	↓	-10.8	-13.06	0.03	93.29±1.4	155.2±3.98	↑	61.9	66.35	0.05
Met	14.64±6.27	16.11±2.85	↑	1.47	10.04	0.29	11.73±0.17	27.25±0.7	↑	15.52	132.3	0.05
Phe	42.57±5.25	48.41±12.45	↑	5.84	13.72	0.03	63±0.94	101.55±2.6	↑	38.55	61.2	0.05
ASA	0.06±0.03	0.09±0.03	↑	0.02	39.52	0.01	0.00001±.000001	0.01±0.0002	↑	0.009	930	0.03
Tyr	47.3±8.52	63.62±23.54	↑	16.32	34.5	0.002	28.23±0.42	46.24±1.18	↑	18.01	63.8	0.05
Asp	27.05±3.96	32.18±5.76	↑	5.13	18.96	0.001	33.62±0.5	45.13±1.15	↑	11.51	34.24	0.05
Ala	159.6±34.21	163.2±37.12	↑	3.6	2.26	0.71	331.89±4.98	563.48±14.47	↑	231.59	69.78	0.05
Arg	17.29±7.48	16.3±5.25	↓	-0.99	-5.73	0.59	43.45±0.65	34.96±0.89	↓	-8.49	-19.54	0.05
Cit	10.79±2.65	12.9±3.23	↑	2.11	19.56	0.01	6.68±0.1	11.98±0.3	↑	5.3	79.34	0.05
Gly	191.1±37.94	272.1±47.69	↑	81	42.39	<0.001	186.78±2.8	250.15±6.42	↑	63.37	33.93	0.05
Orn	75.22±14.49	83.27±19.13	↑	8.05	10.7	0.1	97.29±1.46	152.24±3.91	↑	54.95	56.48	0.05
Glu	271.7±34.96	344.2±65.25	↑	72.5	26.68	<0.001	102.86±1.54	149.73±3.84	↑	46.87	45.57	0.05

## Discussion

Measurement errors can occur when dense molecules in the sample content affect the analysis. Tandem MS (LC-MS/MS) is commonly used as a reference method for newborn [8] screening. Although this is a reference method, carbohydrates, lipids, phospholipids, proteins, anticoagulants, and drugs in the sample content may cause analytical errors [9] [10]. Commonly used vitamins have also been shown to cause interference [11] [12]. Reported analytical errors due to biotin-containing supplements have indicated a need to revisit measurement methods [13] [14]. The seriousness of this issue has been noted by the FDA [15]. Other vitamins, such as biotin, also have the potential to interfere with measurement methods. This study investigated the effect of vitamin K1, which is mandatory for routine use, on newborn screening tests.

When heel blood samples taken 12 hours after birth were compared, the observed carnitine level changes may have been due to nutrition and the increased metabolic activity of the infants. To rule out this effect, the deviation rates of tests in which vitamin K1 was dripped back into the heel blood samples taken before vitamin K1 injection (in vitro application) were calculated. The occurrence of similar positive or negative interferences after the in vitro and in vivo studies suggested that carnitine levels were affected by vitamin K1. In two studies conducted with in vitro and in vivo interference tests, C0, C2, C3, C4, C4DC, C5, C5OH, C6, C8, C10, C10:1, C14, C16, C16:1, C18, C18:1, methylglutaryl, phenylalanine, argininosuccinic acid, tyrosine, aspartate, arginine, citrulline, glycine, and glutamine all showed significantly positive increments. Although C5:1, C8:1, methionine, ornithine, and alanine significantly increased in the in vitro analysis, these values only slightly increased in the in vitro study. Furthermore, different rates of deviation were observed in other tests.

It is difficult to predict at which stage interference may occur in the analysis. The thermodynamic interactions between many molecules in the blood, the diversity of which is unknown, may differ across time [16] [17]. Thus, interference can always be considered as a potential cause of incompatibility between laboratory results and clinical findings. It may be possible to detect random measurement error through repeated analysis. However, this is not a solution, as the repeated analysis of a sample containing interference will not prevent interference. In such cases, sample repetition after the source of interference is removed from the blood may be proposed as a solution for accurate measurement. Given that clinicians may know little about interference, it may go unnoticed and thus result in incorrect interventions. The worldwide number of erroneous measurements in newborn screening programs remains unknown [18]. However, it has been reported that there are, on average, more than 50 false-positive results for every true-positive result of newborn screening in the United States [19]. In a previous study, Tarini et al. [20] found a false-positive NBS result for 818 out of 49,959 infants. Furthermore, they declared that there is a significant interaction between gestational age and NBS results. If heel blood collection is not performed carefully (e.g., if blood flows out of the circle or the heel is over-squeezed), incorrect results may occur. Although the error probability of reference methods such as LC-MS/MS is low, studies have shown that the results of these methods can be deviated by the matrix effect [21] [22] [23]. This effect occurs when any molecule in the blood affects the amount of ionization of the measured analyte. A molecule with interferant properties is capable of disrupting the ionization of the analyte [24] [25]. This effect can lead to delayed metabolic disease diagnosis in newborns, causing irreversible sequelae. In particular, the underestimation of phenylalanine or overestimation of biotidinase activity due to undetected interference can lead to delayed diagnosis and treatment.

The results of this study suggest that erroneously high heel blood test results may occur if the sample is obtained immediately after the administration of vitamin K1. False-positive test results can lead to unnecessary hospitalization, high parental stress, and excessive healthcare use and costs. False-negative results, on the other hand, can lead to delayed diagnosis and irreversible sequelae, such as mental retardation. The fact that interferences yield different results between different individuals can be attributed to variability in the thermodynamic interactions of interferences such as large amino acid molecules, carbohydrates, and proteins in the blood [26] [27] [28] [29].

One limitation of this study was that heel blood samples are not typically taken for at least 24–48 hours. Vitamin K1 levels reach a peak at 12 hours, at which maximum interference can be captured. Due to the necessity of vitamin K1 injection immediately after birth, early heel blood collection was required to detect the vitamin K1 interference effect. It would not be ethically possible to conduct such a study, as vitamin K1 injection by waiting 24–48 hours would pose the risk of bleeding.

## Conclusion

In conclusion, the early collection of heel blood samples by healthcare providers may yield erroneous results due to vitamin K1 administration. Until measurement methods that are less susceptible to interferences are developed, the interpretation of newborn screening results should consider the amount of time between vitamin K1 injection and sampling. Although MS is a reference method, the possibility of erroneous results should not be overlooked. In the case of doubtful results, a new sample should be taken and the measurement should be repeated.

## Dodatak

### Ethics approval and consent to participate

Ethical approval was obtained from the University of Health Sciences, Zekai Tahir Burak Women's Health Education and Research Hospital, Turkey (119/2019, date: 23/07/2019) and approval was obtained from the parents of the infants for the study.

### Human and animal rights

No animals were used for studies that are the basis of this research. This research was conducted on humans are in accordance with the Helsinki Declaration of 1975, as revised in 2013 (http://ethics.iit.edu/ecodes/node/3931).

### Conflict of interest statement

All the authors declare that they have no conflict of interest in this work.
